# NGOs' initiatives and grassroots approach for accessing to health care services for the slum people in Dhaka

**DOI:** 10.3389/frhs.2024.1386698

**Published:** 2024-09-19

**Authors:** Mohammad Ismail Bhuiyan, Md. Aminul Haque

**Affiliations:** Department of Population Sciences, University of Dhaka, Dhaka, Bangladesh

**Keywords:** NGOs, initiatives, grassroots approach, access to healthcare services, slum people

## Abstract

**Objective:**

This study holds significant importance as it aims to delve into the impactful NGOs’ initiatives and grassroots approaches instrumental in providing healthcare services to Dhaka's underserved slum people. It focuses on understanding how these factors influence the use and access to health services, which is a crucial aspect for researchers, policymakers, and healthcare professionals.

**Study design:**

This study was meticulously designed, utilizing a comprehensive cross-sectional mixed-methods design. By incorporating qualitative and quantitative data collection methods, we ensured a thorough understanding of NGOs’ initiatives and grassroots approaches to providing healthcare services to slum dwellers in Dhaka, thereby instilling confidence in the validity of our research for the audience.

**Methods:**

A face-to-face interview was used to survey the participants (*n* = 722) using semi-structured questionnaires, following a systematic sampling technique. Four focus group discussions (FGDs) were also conducted with the slum people. Binary logistic regression was performed to know NGOs’ initiatives, roles, and grassroots approach as predictors or independent variables and healthcare services as an outcome or dependent variable. The quantitative data were analyzed using SPSS version 23.0. At the same time, thematic analysis was conducted following Philip Adu's Qualitative data analysis process and Braun and Clarke's six steps of the thematic analysis system, integrating the 11 subthemes with the quantitative findings to highlight the interpretative findings of the qualitative data.

**Findings:**

Major findings revealed that NGOs’ initiative roles and grassroots approach had a significant impact on slum dwellers’ use and access to healthcare services. The initiatives included affordable health services (OR = 22.86, 95% CI = 3.87, 35.00, *P* = 0.01), special health services (OR = 5.63, 95% CI = 3.36, 9.42, *P* = 0.00), engagement of responsible community leaders (OR = 1.72, 95% CI = 1.14, 2.59, *P* = 0.01), distribution of health and medicine items (OR = 1.92, 95% 2 CI = 1.40, 2.63, *P* = 0.01), provision of updated information to slum dwellers (OR = 1.37, 95% CI = .99, 1.90, *P* = 0.05), telehealth and telemedicine (OR = 1.82, 95% CI = 1.55, 2.13, *P* = 0.01), BCC strategy (OR = 1.26, 95% CI = 1.00, 1.57, *P* = 0.05), and doorstep services as NGOs’ grassroots approach (OR = 1.84, 95% CI = 1.00, 3.38, *P* = 0.05). Qualitative findings supported the quantitative findings through 2 main themes and 11 sub-themes, which were integrated with quantitative findings to highlight the interpretative findings of qualitative data.

**Conclusions:**

Health services and other facilities for urban slum people through NGOs’ initiatives and grassroots approaches are highly affordable and practical, special health services with the involvement of special exceptional health professionals, community supportive services, BCC strategies, and doorstep health services may trigger the use and access to health services for slum dwellers. Results suggest and recommend capitalizing and investing in such initiatives and grassroots approaches from the government, policymakers, and donors with NGOs to find accessible, affordable health services for the unprivileged slum people.

## Introduction

Non-government organizations (NGOs) play a crucial role in development and are long-trusted partners with the government in implementing various social development activities in Bangladesh (1). They provide multiple services to reach disadvantaged populations through collaborative and participatory efforts, including primary education and healthcare (2, 3). NGOs have emerged as a vital alternative for making health services accessible to low-income populations (4, 5). Since their inception, NGOs have been providing health services to the underserved and disadvantaged rural and urban slum people in our country. They have undertaken various innovative and initiative efforts by working in slums, including projects to provide water, sanitation, and housing and to improve child survival, early childhood development, nutrition, and health (5, 6). NGOs are vital in extending primary healthcare or essential health services to underserved, remote rural, and urban periphery slum areas (7). They established clinics, operated satellite clinics or mobile health units, and employed community health workers or community service providers to bridge gaps in healthcare access (8, 9). They were the core implementing partner of the second Urban Primary Health Care Services Delivery Project (UPHCSDP) (10). Behavior change communication, outreach, counseling, and marketing activities were undertaken to improve maternal and child health, nutrition, and family planning, including primary healthcare services for the urban poor (11). As a health promotion strategy, NGOs started training to produce community health workers (CHWs) (12, 13). The NGO Gonoshasthaya Kendra (GSK) trained para-professionals to provide primary healthcare. Similarly, the Bangladesh Rural Advancement Committee (BRAC) has taken the initiative to train almost 10,000 female CHWs in caring for selected common illnesses (14).

Bangladesh's urban population is increasing rapidly. The average annual growth rate was 3.19% from 2018 to 2022. The urban population was 52.07 million in 2022, which is expected to reach 92.0 million by 2050 (15–17). With the expansion of urbanization and urban population, the number of slum people also increases (17.58 million in 2022) (15). Bangladesh is committed to achieving set health indicators of the Sustainable Development Goals (SDGs) (18). Urban areas, specifically urban slums, present health conditions that are challenging for the government. Thus, the government is collaborating with hundreds of national and international NGOs to provide health services in Bangladesh (19, 20). The NGO Health Service Delivery Project (NHSDP) of the United States Agency for International Development (USAID) is a flagship program in Bangladesh implemented from 1990 to 2018 to provide essential health services (ESP), maternal child health, and family planning services in partnership with 26 local NGOs (21). In Bangladesh, the mandate for providing primary healthcare in urban areas is the responsibility of the Ministry of Local Government, Rural Development, and Cooperatives within their administrative jurisdiction. It has urban primary healthcare programs to support essential health packages, free health services, red cards for ultra-poor, reduced-cost medicine, and outreach services for slum and floating people for slum people in partnership with 36 NGOs (22). The World Health Organization (WHO) provides medical aid such as vaccination, research support, and other advanced medical technologies for Bangladesh to improve health in urban slums (23). Tuberculosis, malaria, HIV, and AIDS are also taken care of with the financial and technical assistance of the Global Fund. NGOs primarily deliver maternal child health and family planning (MCH-FP) services in urban slums with paramedics and community health workers (CHWs) (24). Functional collaboration was strengthened with city corporations’ urban primary healthcare (UPHC) program for reproductive health: MCH-FP services in the city slums and outside (25).

NGOs emphasized that community-based health workers or volunteers should go door to door in every urban slum and village, providing doorstep services to citizens (6, 26). Satellite clinics were established to provide maternal and child health (MCH) services to the slum dwellers (27–29). Mobile (mHealth) services were also introduced by NGOs in the urban slum context, where people have limited access to health services (30, 31). In case of doorstep service initiatives are unavailable due to health workforce shortage, time constraints, and moving from community to community, then NGOs initiated mobile health (mHealth) services (32). This mHealth service has widened access to healthcare for the slum people (26, 32). Using mobile phones can enhance efficiency and continuity of care during the antenatal and postnatal period, thereby improving the accessibility and delivery of maternal and child healthcare among women (26). Since 2007, BRAC has been implementing some approaches to promote positive Maternal, Neonatal, and Child Health (MNCH) behaviors and practices (33). Manoshi developed key messages for behavior change and applied different means of communication using different BRAC health staff, including the community health volunteers (*Shasthya Sebika*) and community health workers (*Shasthya Karmi*, program organizer) (34). At the community level, the NGOs take the initiative to employ female Family Health Visitors (FHVs) who conduct visits to an allocated number of households. They are responsible for essential health and family planning counseling, doorstep delivery of contraceptives (oral pills and condoms) and oral rehydration salts, and mobilization of women to use satellite clinics and higher-level facilities (35–38). At the Mohammadpur slum area of Dhaka city, under public and private partnership (PPP), a maternity clinic initiated and launched affordable and accessible healthcare services for low-income people. This health project also provided health education, became a referral point for poor patients, and sent complicated cases to specialized hospitals (6, 39).

Grassroots approaches lead to greater participation of members or beneficiaries with more significant support and cooperation from professionals and service providers (40). The grassroots approach to healthcare services mainly focuses on the people who confronted and experienced homelessness or social exclusion, especially low-income urban people or migrant slum people (41, 42). The full realization of grassroots healthcare services to improve people's access to healthcare services and ensure adequate resources for disease prevention is significant. Hence, it is essential to optimize the services of grassroots health facilities (including NGOs) and guide the masses to seek, utilize, and receive medical or healthcare and services reasonably (43). Several studies in developing countries also showed that the grassroots approach of NGOs made healthcare services more effective for low-income people than public and private providers because they are locally based and are more accountable to their communities (44–46). Very little is known from previous research on NGOs’ initiatives and grassroots approaches to healthcare for slum people in Bangladesh. Most of the prior research studied a partial or single aspect of NGOs’ initiatives such as affordability or subsidizing services, BCC strategy, telehealth and telemedicine services, and doorstep services. Therefore, the current study addresses the knowledge gap and covers NGOs’ multifarious initiatives and grassroots approaches to accessing healthcare services for the slum people.

## NGOs’ initiative roles and grassroots approach for accessing healthcare services for slum people: a point of view from South Asian stance

The roots of NGOs can be traced back to various social and political movements that emerged in the 19th century. During this period, rapid industrialization and urbanization led to widespread poverty, labor exploitation, and the marginalization of vulnerable populations. In response to these issues, groups of concerned individuals began organizing themselves to advocate for social reforms and humanitarian initiatives, giving birth to the early form of NGOs (14, 47). Over the past few decades, citizens in low-income countries have established increasing numbers of NGOs to serve unmet local needs. Poverty, disasters, war, health, and other misfortunes provided grounds and reasons for NGOs to flourish in low-income countries. Most NGOs in developing countries have their bases in rural areas and urban slums, often not served at all or minimally served by the existing government service structure (26). An estimated 41.8% of Mumbai's population lives in urban slums (48). The health condition of the slums in both Mumbai and Delhi is worsened by a high degree of poverty and low access to resources combined with low living conditions (48, 49), which leads to a greater degree of infections, disease, and health conditions. The country (India) has a mixed healthcare system, with approximately 10.0% of hospitals being government-run and the rest operated by private for-profit sectors or charitable organizations as well as NGOs (49–51). In the Panjrapole slum of Mumbai, 31.0% of respondents consulted private/NGO doctors due to dissatisfaction with the quality of care at government/public hospitals (49, 50). In 2022, 48.0% of the total urban population of 10 big cities in Pakistan live in slum areas (52), and NGOs are the significant contributors to the choice of healthcare services (53). The government of Indonesia launched the National Health Insurance (NHI) in 2014 to cover people with low financial status, including slum dwellers (54). NGOs in Indonesia have played a crucial role in mobilizing the resources of the urban people in low-income communities (55). In developing countries such as the Philippines, up to one-third of healthcare services are provided by non-profit organizations (NPOs) and NGOs. NGOs, being small-scale and flexible, help poor communities provide services where government assistance is usually minimal and ineffective (56, 57).

The above literature and their discussion and illustrations denote and uphold NGOs’ presence and rigorous functions in the urban slum areas of India, Pakistan, Indonesia, and the Philippines, where government services are minimal, inadequate, and unavailable. Bangladeshi NGOs administer a network with static and satellite clinics, covering 20 million Bangladeshi areas where government services are largely inaccessible. In addition to clinic-based services, many NGOs support female community workers, called depot-holders, who provide health commodities, BCC strategy, and referral services to NGO clinics (44). NGOs generally work with disadvantaged and unprivileged people at the grassroots level. Their service provider is more concentrated and attached to the urban slum disadvantaged people than the government employees and healthcare providers (8).

The current study seeks imperative empirical data on NGOs’ initiatives and grassroots approaches toward accessing healthcare services for the slum people in Dhaka. To answer the research question or achieve the research objective, some metatheories, especially Andersen's Behavioral Model of Health Services Use, were used as the most appropriate model for understanding and predicting the use and access to health services for the slums in Dhaka city. The manuscript has several parts. The introductory part consists of contextual discussion, a statement of problems, and a scenario of healthcare services for NGOs in South Asian slums, highlighted with relevant literature. The next part contains the methodology section, which includes study design, study settings, study population and sampling, variables and measurement of the study, and data collection and analysis process. The subsequent section comprises the results (findings) of the study, which represent the answer to the research questions/objective. The next chapter is part of the discussion section, where significant findings reveal similarities and dissimilarities of existing literature and limitations, as well as the scope of the study and significant implications suggested in this section. Finally, the conclusion implies the study's significant findings and recommendations for further initiatives or steps from the government and policymakers.

## Access to health services: a theoretical overview

**Figure 1:**
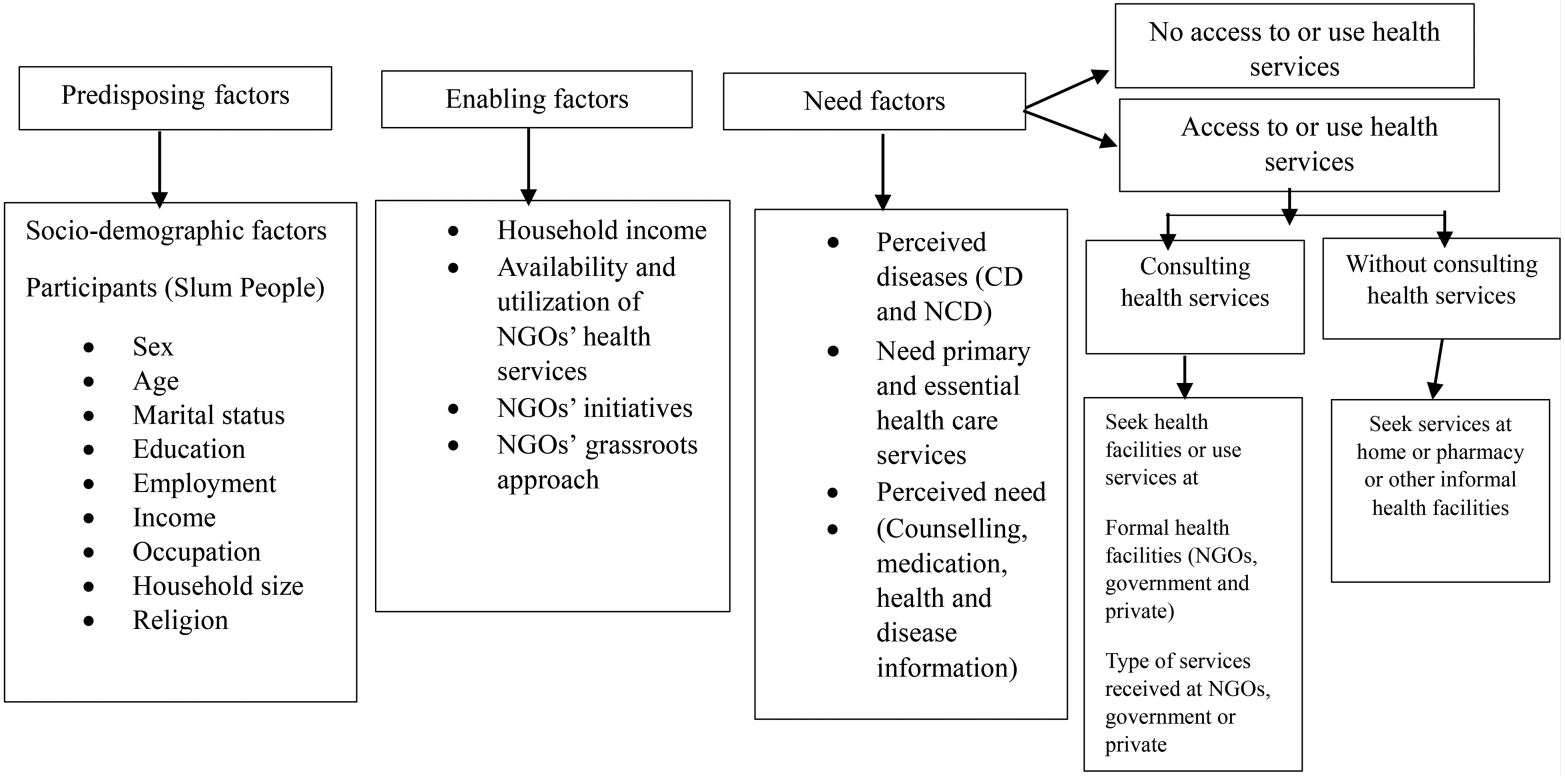
The conceptual framework of this study adopted from Andersen's Behavioral Model of Health Services Use (59, 60).

Theories attempt to explain phenomena logically and meaningfully, often following narrative structures (58). The theoretical and conceptual framework describes the research avenue and grounds it firmly in theoretical constructs. Andersen's Behavioral Model (59, 60) for Health Services Use provides a theoretical structure to understand access to and utilization of health services and recognize the factors that impact an individual's and community's decision to use or not use existing health services. According to the model, access to and use of health services is determined by three dynamic factors: predisposing, enabling, and need factors. Predisposing factors can be social and demographic characteristics such as sex, age, marital status, education, income, occupation, community or ethnicity/race, attitude, and belief, indicating and increasing one's need for health services. For example, an individual or community who believes health service is an effective treatment or health services are needed to cure or prevent diseases he/she/they are more likely to seek care. Andersen's enabling factors are the main factors that facilitate or assist people to use or access health services (e.g., resource availability or service affordability) (59, 60). The enabling factors are, for instance, household income and support, initiatives to use services, access to free services or insurance or subsidizing services, spatial or geographical nearest, and availability and affordability of those services. Enabling factors facilitate and impede one's or community's access to and use of health services. Need factors represent self-perceived and actual need for healthcare services. Therefore, need factors denote or push individuals/communities (patients) to what type of health services they do or do need for their ailments or diseases. The predisposing factors of this study indicate the sociodemographic characteristics of participants, who comprise the study population of the study settings (Figure 1).

## Methods

### Study design

This study used a cross-sectional mixed methods design to collect both quantitative and qualitative data. A face-to-face interview was conducted using a semi-structured questionnaire to obtain quantitative data from 722 respondents, of whom 329 were males and 393 were females. Qualitative data were administered through focus group discussions (FGDs) with the help of Adu's (61) qualitative data analysis process and Braun and Clarke's (62) six principles of thematic analysis system and following phenomenological study.

### Study setting

**Map 1:**
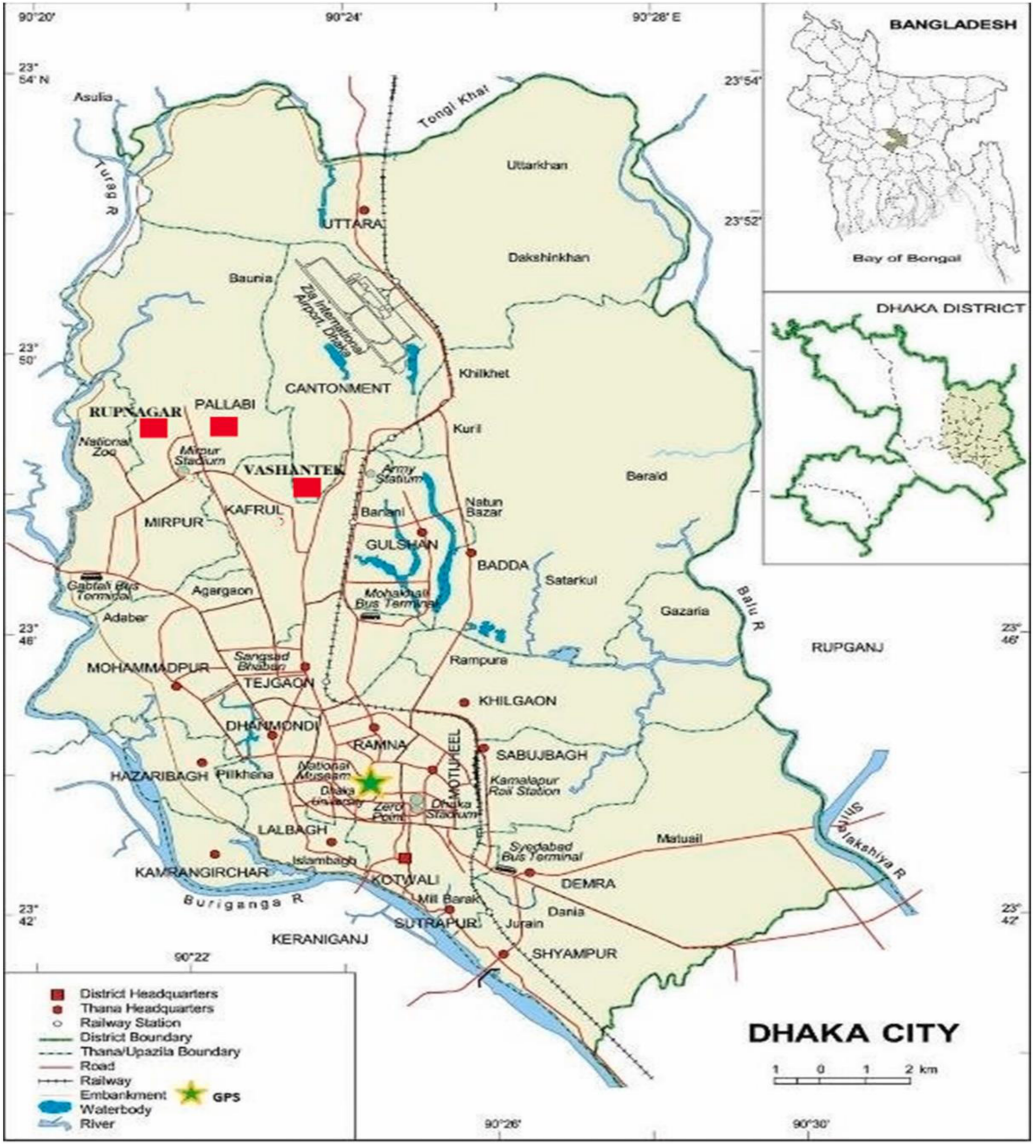
Study area map, including both Dhaka North and Dhaka South City Corporation (map source: Internet).

The slums of three Thanas Pallabi, Rupnagar, and Vashantek Thanas from the Dhaka North City Corporation were selected for this study (Map 1). Thana is the second level of the administrative unit from below in Bangladesh. The Dhaka division and district were selected purposively as Dhaka, the capital of Bangladesh, was considered to have more slums than the country's other cities. Then, the Dhaka North City Corporation was chosen randomly, and the three Thanas were considered based on the location of the larger slums (63).

### Study population and sampling

This study used a sampling formula where *z* = 1.96 set at 95% confidence level; *p* = 0.5, proportion of slum people used NGO's health services; *q* = 0.5; margin of error = 0.05; and design effect = 2.0. Using the formula, the sample size was 768 (384 × 2 = 768). The sample was distributed among the selected three slums using probability proportionate sampling. Several males and females were taken into consideration. The sample frame was prepared, and the required number (768 households) of sample households was selected from the sample frame using systematic sample techniques. Finally, 722 respondents (322 males and 393 females) successfully attended the interview with a response rate of 94.0% (Table 1). Reasons for exclusion or non-responses to the interviews were deaths, divorce, mobility to other places, separation, and absence from home. Respondents were between the ages of 15 and 74 years, and they were able to provide information and were included in the study. Four focus group discussions (FGDs) were conducted to collect qualitative data. This study followed the door-to-door recruitment process, considering the nature of the study and its respondents. Variables included in the study are presented in Table 2.

**Table 1:** Distribution of study population and respondents.

Study Thana	Estimated population in each Thana	Estimated sample size	Collected sample size
Pallabi	4,238	228	216
Rupnagor	2,818	164	158
Vashantek	7,390	376	348
Total	14,446	768	722

Qualitative: focus group discussion (FGDs) 4 × 10 = 40 people.

**Table 2:** Variables, operational definitions, and their level of measurement.

Variables	Indicators	Operational definitions of the variables	Level of Measurements
NGOs’ role-related variables/enabling resources (based on Andersen's model)	Initiative roles or step	Quick and high priority-based services for quality and rapid services	Nominal
	Affordable services	The capability of bearing the expenses of services, medicines, and products	Nominal
	Special services	Special health services focus on priority and distinctive basic services	Nominal
	Community supportive services	Slum community leaders perform and provide health services to the community through concerted efforts on behalf of NGOs	Nominal
	BCC strategy	Communication strategies to promote positive health behavior through interaction and intervention	Nominal
	e-technology	Health services are provided and brought to the community through mobile and social media.	Nominal
	Counselling	Trained and professional people provide health services for Essential MCH, reproductive, and adolescent health.	Nominal
	Updated information	Alert people with new and regular information	Nominal
	Grassroots approach	Meticulous, thorough, and wide-ranging services are provided with the involvement of CSP/CHW or community peer group for each individual and household of the slum	Nominal
	Doorstep services	Health service delivered at home	Nominal
	Provided 24 h services by CSP	Deliver services round the clock	Nominal
	Access to health services	The ability to use health services when and where they are needed	Nominal

### Data collection and analysis process

**Figure 2:**
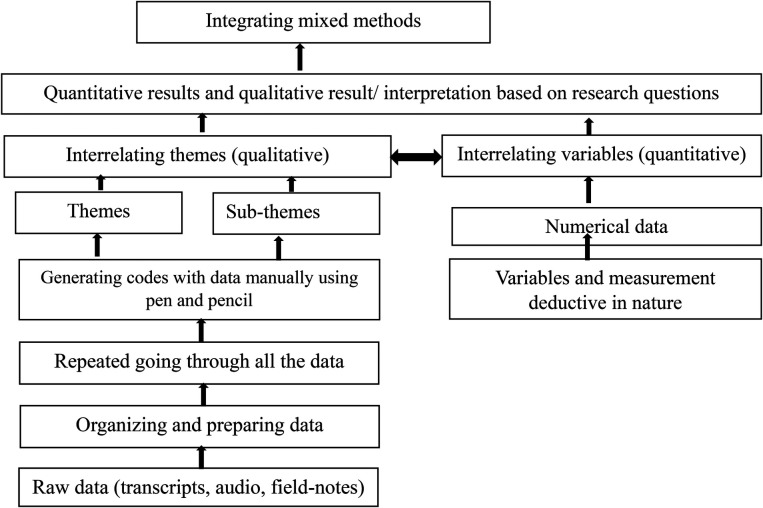
Processes or stages of mixed methods data analysis and stages of processing qualitative data analysis.

A total of four (two males and two females) masters-level students with experience in field-level data collection were recruited as research assistants and were engaged and trained by the researcher. The researcher engaged and trained research assistants before the pretest and final survey. The questionnaire was pretested with different slum people and slum settings. Some critical issues, such as health service-related questions, wording of the questions, and time management, were learned from the pretest. After adjustment of the data collection instruments, a face-to-face interview was conducted at the households or a convenient place of the respondent's household from August 2021 to September 2021. Almost 25–30 min was required to complete each interview. A total of four FGDs were conducted, each consisting of 9–11 participants. The researcher conducted each session as a moderator, while research assistants performed as the notetakers. Descriptive statistics was used to present the socioeconomic characteristics of the respondents. Pearson's chi-square test was used to know the factors associated with NGO's initiatives and access to healthcare. Finally, a binary logistics regression model was used to determine the factors associated with NGO's initiatives using Statistical Package for the Social Sciences (SPSS) version 23.0 and Microsoft Excel 2019. The results were presented as odds ratio (OR) with a 95% confidence interval (95% CI). We used *P*-values to show the probability of error while rejecting the test and presented the odds ratio to express the effect size. Qualitative data were transcribed, and thematic analysis was conducted with the help of Adu's (61) qualitative data coding and analysis and Braun and Clarke's six-step thematic analysis (62) related to the research question/objectives (Figure 2).

### Ethical issues

This study seriously followed and addressed ethical matters. Before starting and conducting the data collection process, an approval letter was asked by the corresponding author and obtained from the chairman of the department of the university. Throughout the study, the researcher and his team always considered ethical principles established for human participants under the Helsinki Declaration. Verbal consents were also taken from the participants and informed them about the purpose of the study before starting the interview. Participants were assured about the anonymity of their names and identities and informed about using their data only for academic purposes. Participants’ identities were encoded with specific numbers for the betterment of both data collectors and participants. As the data collection process of this study was conducted during COVID-19, some specific precautions and safety measures such as using masks, keeping sanitizer with both data collectors and study participants, and physical distancing were implemented. Social gatherings are also avoided during the data collection process.

## Results

### Sociodemographic characteristics of the respondents

Table 3 demonstrates the sociodemographic characteristics of the respondents. The study population included 722 respondents from the three Thanas of seven slums, where most participants were female (54.4%), and the minority was identified as male respondents (45.6%). Age ranges (25–44) covered most respondents (57.1%). Most of the participants (92.4%) were married, and the majority (39.1%) of the respondents were part of “no education,” which meant illiterate. A greater portion of the respondents (34.6%) were housewives involved in household chores, and a vital proportion of participants (39.2%) were dependent on the income of their husbands, sons, and wives of the households.

**Table 3:** Sociodemographic or background characteristics of the respondents (*n* = 722).

Variables	Frequencies	Percentage (%)
Sex		
Male	329	45.6
Female	393	54.4
Age		
15–24	118	16.3
25–34	223	30.9
35–44	189	26.2
45–54	104	14.4
55–64	65	9.0
65–74	23	3.2
Marital status		
Married	672	93.1
Unmarried	4	0.6
Separated	12	1.7
Widow/widower	34	4.7
Educational qualification		
No education	282	39.1
Primary	242	33.5
Secondary incomplete	139	19.3
Secondary school certificate (SSC)	24	3.3
Higher secondary certificate (HSC)	27	3.7
HSC +	8	1.1
Employment status		
Employed	188	26.0
Unemployed	40	5.5
Self-employed	184	25.5
Day labourer	60	8.4
Housewife	250	34.6
Income (BDT)		
Dependent	283	39.2
<5,000	78	10.8
5,000	24	3.3
5,001–10,000	189	26.2
10,000+	148	20.5
Total	722	100.0

**Figure 3:**
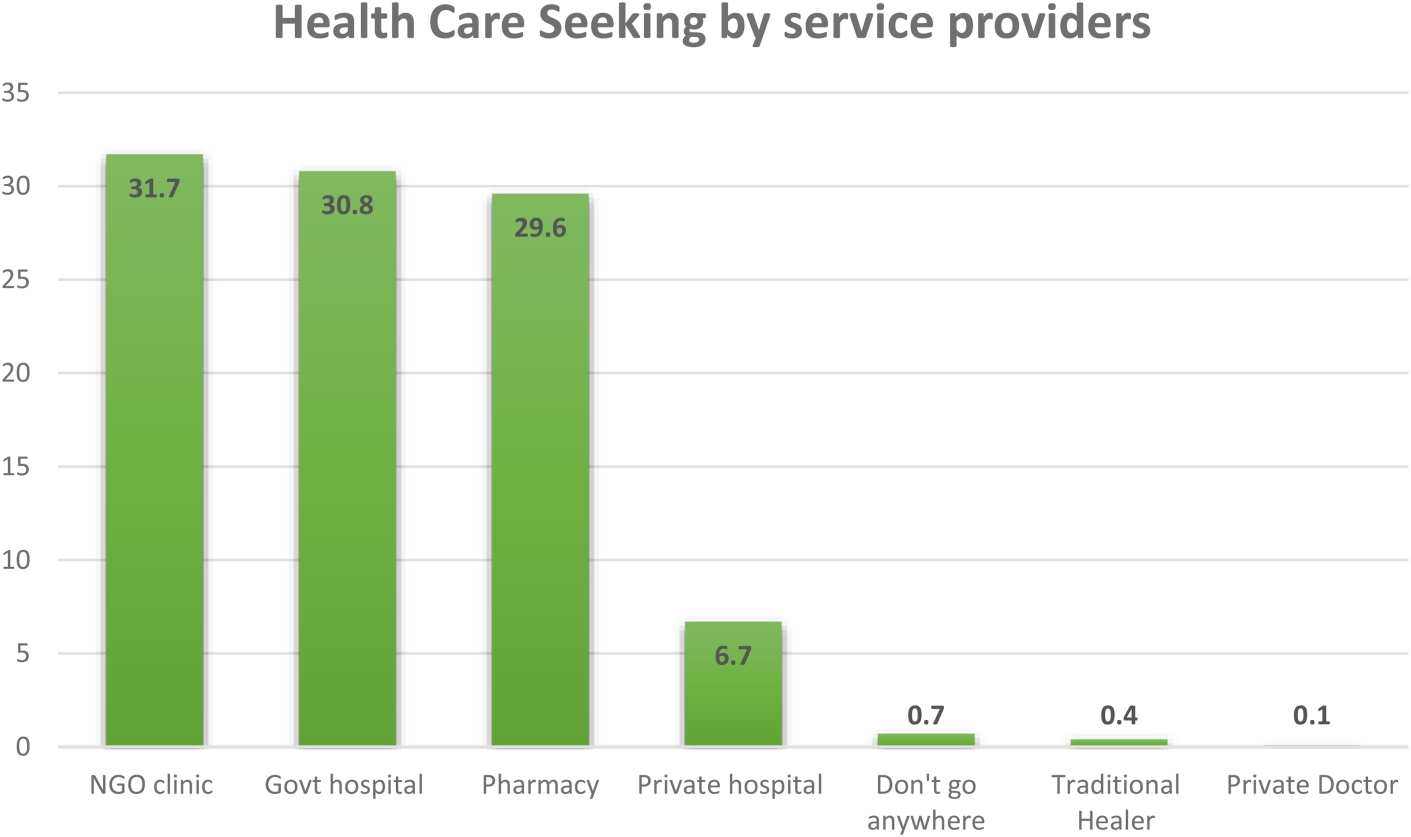
Visiting healthcare centers by seeking health services.

Figure 3 illustrates the study's respondents’ visits to the healthcare centers. Here, the figure is shown through the usage of multiple responses. One patient/respondent said that she/he might use or go to government, private, and NGO healthcare centers simultaneously for her/his necessary healthcare services. Participants (30.9%) stated that they would go to government hospitals. A small number of respondents (6.7%) shared that they went to private hospitals or healthcare centers to receive healthcare services. Most participants (31.7%) of this study said they used or went to NGO healthcare centers/clinics for health services. A significant number of respondents (29.6%) reported that they experienced and experimented with healthcare services from their nearby pharmacies.

Table 4 presents the NGO's initiatives and grassroots approach to accessing healthcare services for the slum people using a multivariate logistic regression model. Initiatives and grassroots approach were the two main predictor variables, and these two variables had 11 sub-variables, which demonstrated the association with the outcome variable (visiting or access to NGO healthcare services). The study showed that the NGOs’ initiatives (seven initiatives) and grassroots approach (doorstep services) were significantly associated with access to healthcare services. The study found NGOs’ initiatives and grassroots approach to affordable services (OR = 22.86, 95% CI: 3.87–35.00, *P* = 0.01), special health services (OR = 5.63, 95% CI: 3.36–9.42, *P* = 0.000), doorstep services (OR = 1.84, 95% CI: 1.00–3.38, *P* = 0.05), engaging responsible community leaders (OR = 1.72, 95% CI: 1.14–2.59, *P* = 0.01), distribution of medical and medicine items (OR = 1.92, 95% CI: 1.40–2.63, *P* = 0.01), update slum dwellers with updated information (OR = 1.37, 95% CI: 0.99–1.90, *P* = 0.05), e-health technology (OR = 1.56, 95% CI: 1.28–1.99, *P* = 0.00), and BCC strategy (OR = 1.26, 95% CI: 1.00–1.57, *P* = 0.05) were significantly associated with dependent variable access to healthcare services.

**Table 4:** NGOs’ initiatives and grassroots approach for accessing healthcare services for slum people.

Independent variables	B^a^	S. E	*P*- value	Exp (B)	95% CI for Exp (B)
Affordable health packages: Yes (Ref Category: No)	3.13	0.906	0.01	22.86	3.87–35.00
Special health services: Yes (Ref Category: No)	1.73	0.263	0.00	5.63	3.36–9.42
Regular site visiting: Yes (Ref Category: No)	4.47	1,799.3	0.99	87.1	0.00-
Doorstep services: Yes (Ref Category: No)	0.61	0.311	0.05	1.84	1.00–3.38
Counseling services: Yes (Ref Category: No)	0.39	0.273	0.15	1.48	0.87–2.53
Engaging responsible community leaders: Yes (Ref Category: No)	0.54	0.210	0.01	1.72	1.14–2.59
Distributing leaflets, brochures, etc.: Yes (Ref Category: No)	0.65	0.160	0.01	1.92	1.40–2.63
Updating slum people with updated health information: Yes (Ref Category: No)	0.32	0.165	0.05	1.37	0.99–1.90
Free services for red card holders: Yes (Ref Category: No)	1.86	337.9	0.99	6.39	0.00–2.98
Telehealth and telemedicine: Yes (Ref Category: No)	0.46	0.108	0.00	1.56	1.28–1.99
BCC strategy: Yes (Ref Category: No)	0.23	0.113	0.05	1.26	1.00–1.57

B^a^, regression coefficient slope (*β*), (*P < *0.000; *P* < 0.01; *P* < 0.1; *P* < 0.05), Exp (B) = OR, 95% CI for odds ratio (exp B^b^).

Table 5 describes the special incentive healthcare packages of NGOs. Special incentive healthcare packages are part of the NGOs’ initiative roles. Incentive packages of healthcare services of NGOs ensured better access to healthcare services for slum dwellers. The majority (27.1%) confessed that FP services were the most proposed incentive package of healthcare services NGOs offer for their slum people. Both service providers of NGOs and respondents of slum people shared their experiences that these FP services continue to operate upon the capable or eligible reproductive age group (15–49). The second most crucial incentive package of health services NGOs provide is various blood tests (22.7%). Delivery care, both normal and caesurae (19.1%), became the third most incentive package of healthcare services offered by NGOs for the participants. Respondents (14.0%) reported that they would receive antenatal care as the most initiative package of their healthcare services from an NGO. Additionally, 8.9% of respondents mentioned they would enjoy discounts on their required health services.

**Table 5:** Special incentive healthcare packages provided by NGOs.

Healthcare services	Number of respondents (%)
Delivery services^a^	152 (19.1)
Family Planning services	215 (27.1)
Ultrasonogram	65 (8.2)
Blood test	180 (22.7)
Antenatal care services	111 (14.0)
Discount offered	71 (8.9)

^a^ Multiple responses.

### Qualitative part

**Figure 4:**
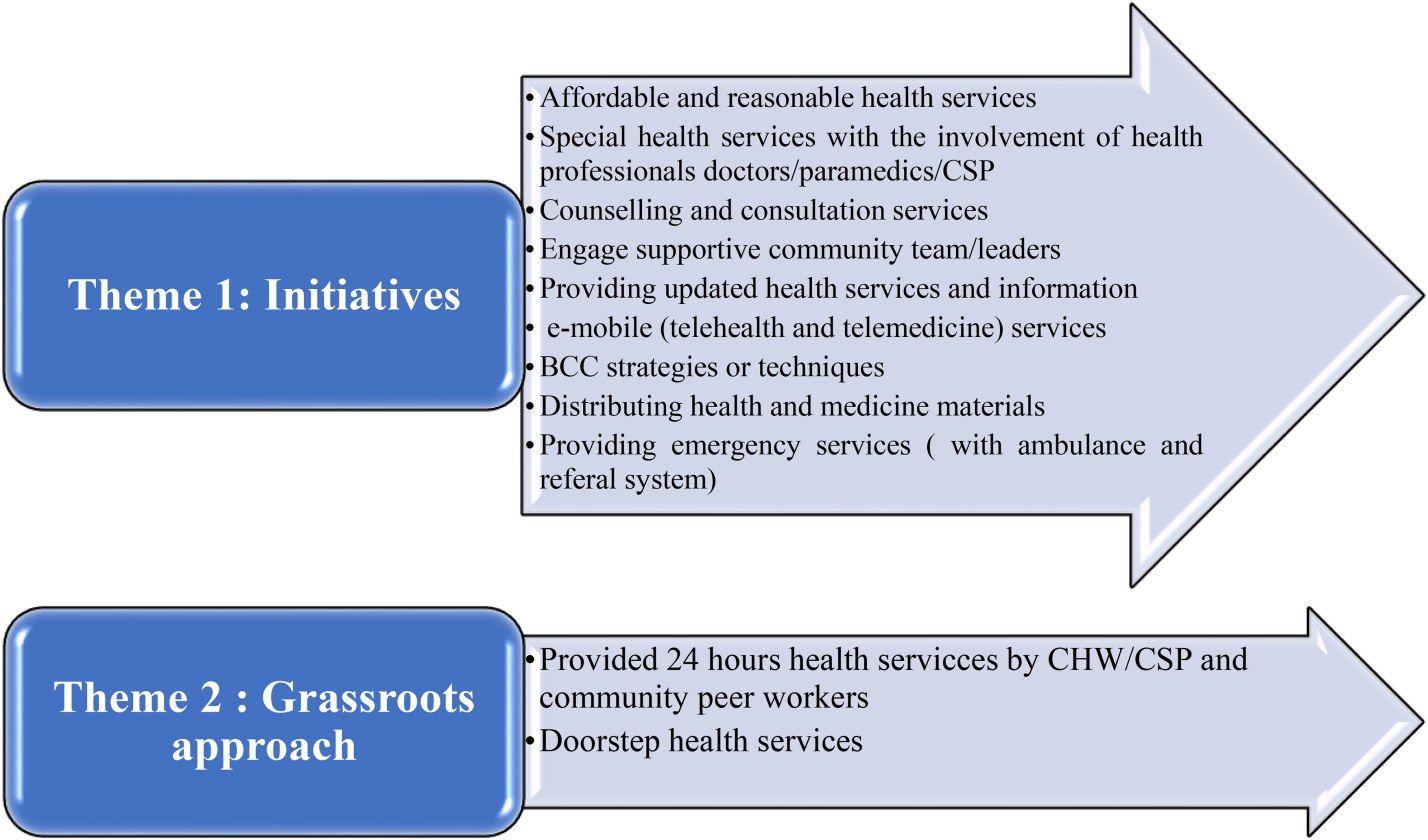
The themes and subthemes of the qualitative study.

A thematic analysis was used to present the qualitative data. Two main themes and 11 sub-themes emerged from the collected data. These themes and subthemes were coded and analyzed using Adu’s and Braun and Clarke's thematic analysis process and principles, as shown in Figure 4.

### Theme 1: initiatives

Respondents shared their experiences and opinions about the initiatives of NGOs in providing healthcare services. According to their opinions, such initiatives of NGOs brought about drastic changes in the arena of health and the usage of health services for their slums and slum people. These initiatives have nine subthemes: affordable and reasonable health services, special health services with the involvement of health professionals, counseling and consultation services, engaging supportive community team/leaders, providing updated health information, e-mobile (telehealth and telemedicine), BCC strategy/techniques, distributing health and medicine materials, and providing emergency services either with their own health professionals or referral system.

#### Affordable and reasonable health services

Respondents expressed that the NGOs sometimes undertook initiatives to ensure affordable and reasonable health services for our slum dwellers. They provided some affordable and reasonable health services for our people. Most participants described that expanded programs on immunization (EPI) services, FP services, and some tests such as blood tests and pregnancy tests became affordable for them. The researcher and others concerned with this study observed various personal and non-personal communication, such as campaigns with community supportive teams, hanging banners, and *miking* conducted around the slum areas for free and reasonable health services.

Responsible persons of NGOs or community service providers (CSP) in our locality or area sometimes inform us about NGOs’ initiatives on low and affordable service packages. They provide our services at low or reasonable prices. (A veteran service beneficiary)

#### Special health services with the involvement of health professionals such as doctors, paramedics, and CSP

NGOs have been providing a range of specialized healthcare services in the slum areas. These include Maternal and Child Health (MCH) services, hepatitis B programs, special eye care services, vitamin A capsule campaigns, and sanitary and hygienic services. The involvement of special health professionals, such as doctors and paramedics, in these services has been crucial in addressing the unique healthcare needs of the slum population.

When any health services or program decided to launch or conduct our slums, we immediately got informed by our CSP or community supportive team or leaders over the phone or in meetings. Those who were considered patients and suffered from related diseases or morbidity received services from the ongoing program or campaign of NGO. (An MCH patient)

#### Counseling and consultation services by nurses, paramedics, and CSP/CHW

Respondents of this study expressed that NGOs’ counseling services took place through NGOs’ static clinics, satellite clinics, and CSP. Sometimes, counselors visited slum areas to advise and instruct on different health services. The study found and observed that MCH, FP services, and adolescent and reproductive services were conducted through counseling.

Once or twice a week, a female counselor, a female counselor visited our slum area from the respective NGO, and a female counselor from the respective NGO visited our slum area. We also gained counseling facilities from our paramedics and CSP. However, the counselor who visited us was good at counseling our health services. We found many guidelines like FP services, EPI services, ANC and PNC services, health and nutrition services, and reproductive and adolescent services from the counselor. (A young mother and health beneficiary)

#### Engage supportive community team/leaders

NGOs engaged responsible community teams/leaders in respective slum areas. Most of these leaders were female, and they provided us with early information about healthcare services. It was observed that every slum has an NGO's community-supportive team, which was employed with different lucrative offers and logistic support from NGOs. Slum people derived constant and updated information from their community-supportive team.

We immediately got information about health services, other services, or news from our community leaders. During COVID-19 or before the onset of COVID-19, our community leaders always played a pioneering role in doing our best. Most of the NGOs entered and welcomed to the slums with the aegis of our slum community leaders. (A middle-aged MCH and an FP beneficiary of the NGO)

#### Providing updated health services and information

Participants described that NGOs initiated to provide them with regularly updated information through CSP and community supportive leaders. For this, NGOs provided mobile phones to CSPs to convey updated health information to their respective slum dwellers.

We receive all updated information from our community leaders or CSP. If any information or new health services or packages are offered, soon we get this information from our leaders or CSP and such message is publicized among the people in our slum. During COVID-19, we frequently got health-related information from NGOs to protect us from COVID-19. (A maternal and FP patient)

#### e-Mobile (telehealth and telemedicine) services

Respondents discussed the revolution of health services through e-Mobile and e-Health. Before COVID-19, telehealth and telemedicine health services were meagerly served, but during COVID-19, when all direct healthcare services were hindered and stopped, telehealth and telemedicine became a vital and popular way of receiving persistent health services from NGOs. Especially telemedicine has become a very inevitable service for slum dwellers. The study finds any emergency and critical conditions of a pregnant mother, elderly people, and neonatal complications are immediately resolved through telehealth and telemedicine.

When COVID-19 began to spread, all direct health services through static and satellite clinics almost decided to stop serving in our slums. Authority and persons from NGOs assured us of continuing health services through mobile. Hence, they provided our CSPs with mobiles and continued for 24 h during COVID-19. Specifically, NGOs emphasize providing MCH, TB, and COVID-19 services through telehealth and telemedicine. (A delivery and mother of the neonatal patient)

#### BCC strategies/techniques

Participants vastly shared and discussed their experiences with BCC strategies of NGOs. They were happy with the BCC strategies, which helped them change their attitude and adjust to their healthcare behavior. They learned about BCC strategies from messages CSP or CHW, such as pregnancy care/taking rest and cleanliness, birth preparedness, safe delivery, and postpartum care with neonatal health. Moreover, posters and stickers about the danger signs of pregnancy, the eight newborn danger signs, post-delivery period, the danger signs of high fever, severe headache and blurring of vision, and hemorrhage or excessive bleeding gave them profuse knowledge and made them cautious about health and illness.

It was surprisingly a new experience for me when I came to know and observe the pictures of dangerous signs of a pregnant mother, marriage of a young/adolescent girl, a skinny infant with a young married girl, convulsion and hemorrhage picture of a pregnant mother, and NGOs. I gradually became familiar with BCC strategies and learned many things from the paramedics and NGO CSPs about caring for a maternal mother and her newborn baby. I also learned about the devices of FP services. (A maternal and FP patient)

#### Distributing health and medicine materials

Respondents of this study spontaneously discussed NGOs’ initiative steps for distributing health and medicine materials. Initiatives to distribute health materials encouraged them to use health services. Researchers and research assistants also noticed that NGOs provided slum dwellers with hand sanitizers, masks during and after COVID-19, brochures, pills, vitamins, and paracetamol tablets and syrup.

Receiving health and medicine materials like leaflets, brochures, pregnancy strips, FP devices, hand wash, and hygienic materials made us more vibrant and curious towards NGOs’ health services. Sometimes, training arrangements, meetings with us, and distributed materials and gifts attracted us greatly to their services. (A community leader and service beneficiary)

#### Providing emergency and referral services

Participants confessed that the NGOs’ people are always linked with their community service providers (CSPs) and community leaders to provide them with updated information and emergency health services. Patients with essential healthcare services, communicable diseases, or non-communicable diseases get direct services or referral services from the NGOs’ people.

Getting or finding emergency or immediate health services from NGOs was beyond our imagination and thoughts. One day, my daughter-in-law felt severe pain in her abdomen during her first pregnancy. I was alone at my house and didn’t think about what I should do with her right now. Then I rushed to our community services provider (CSP), but I could not find her in her room, so I informed community support leaders about my daughter-in-law’s condition. They at once communicated with NGO people over the phone and told them about the severity of the pain. NGO people told us to bring my daughter-in-law to their hospital, which was not far away from our slum. We instantly took her to this small NGO hospital and got a new baby after two hours of attempts of all concerned health professionals. (An experienced slum dweller and mother-in-law of a young delivery patient)

### Theme 2: grassroots approach

#### Provided 24 h services by CHW/CSP and community peer workers

NGOs engage community health workers (CHWs), community service providers (CSPs), and community peer health workers to provide 24 h health services for slum dwellers. These CHWs/CSPs and community peer workers were chosen and appointed from the respective slum areas to ensure the grassroots-level health services of the slums.

Our community service providers, health workers, and peer workers became our constant and remedial fellows and friends for providing 24-hour health services for us. During COVID-19, when government health services were insufficient, and most NGOs at service abstained from visiting our slums and stopped providing health services, NGO authorities decided to offer our services within 24 h through our local CHW and peer workers (An MCH health service beneficiary)

#### Doorstep/knocking at the door services

Participants said the doorstep healthcare services were a remarkable and beneficial step for NGOs. NGOs’ paramedics, community service providers (CSPs), and community health workers (CHWs) provided doorstep health services. CSP and CHW are providing constant health services for the slum people because they live in slum areas. Paramedics provided services through satellite clinics and visited slum households for doorstep services. The study observed that service providers of satellite clinics and CSP/CHW were very influential in grassroots-level services because both service providers have strong and close links with the slum dwellers.

We got much benefit from doorstep health services. Our women and pregnant women received many instructions from paramedics and CSP/CHW before and during their pregnancies. Paramedics and CSP advised and instructed our adolescent girls and young and newly married couples about using senora/pads and condoms during intercourse. Both have advised the new couple to use contraceptive devices to avoid or prevent unwanted pregnancy. (Patient of NGOs’ multi-health services).

## Discussion

Health is a fundamental and constitutional right and one of the crucial priority sectors of the government. There has been increasing recognition that a healthy population is essential to economic growth and prosperity. To attain universal health coverage, equitable access to healthcare services must be ensured irrespective of gender, including disability and marginalized populations (64, 65). However, most slum dwellers are not served well by all the layers of health facilities from the top entities of the state except a few national and foreign NGOs (25, 66, 67). Many barriers slum dwellers face in accessing quality health services because of a lack of availing initiatives (68). Existing literature from the scenario of Bangladesh's slums and South Asian and Asian countries’ slums demonstrated and indicated the common barriers, limitations, and deprivations of getting healthcare services from both the public and private ends. Previous studies also plainly presented the challenges in coordinating two ministries of Bangladesh, which deprived urban poor people (e.g., slum dwellers) of finding, availing, and reaching health services (6). Since Bangladesh is committed to providing essential healthcare services to its entire population, many public healthcare facilities are officially free or available at a minimum fee (69). Unfortunately, these services are still unreachable, unavailable, and underutilized in Bangladesh for various reasons, especially for urban poor people (e.g., slum people), some of which are already mentioned. Some facilities, along with initiative steps and grassroots level services from the large and small number of NGOs and GO- NGOs/PPP health projects, ensured better use and access to healthcare services for the urban areas, particularly in slums and squatters settled in Dhaka (70). The significant findings and themes from integrating mixed method study are discussed below.

### NGOs’ initiatives and grassroots approach worked and helped like a super aid and super benediction services for the slum people

The urban slum dwellers have been categorized as “hardcore” and “absolute” poor based on their household incomes and vulnerability. Their purchasing capacity, level of expenditure, living standard, lifestyle, and health condition are deplorable (71). Urban slum people are heavily pushed by migrant people in Bangladesh (72, 73). Hence, such destitute, impoverished, homeless, and socially excluded people always look for and depend on financial assistance and other initiative roles and grassroots-level services of government, donors, and NGOs. Most slum dwellers are informal workers who desperately need urban services while living in horrible conditions (74–76). NGOs and people of these NGOs cordially, cooperatively, and desperately engaged in initiatives and grassroots-level services for slum people. They could be significant in organizing slum dwellers to enjoy and utilize amenities (77). Participants (both quantitative and qualitative) of this study stated and mentioned some initiative roles (affordable and reasonable services, providing special health services with the involvement of health professionals, Engaging responsibly community leaders/community supportive services, e-mobile/telehealth and telemedicine services, distribution health and medicine items, BCC strategy/techniques) and grassroots approach (providing 24 h health services with CHW, doorstep services) of NGOs greatly influenced slum dwellers to use and access to healthcare services. Fair and feasible prices of services ensured better use and access to healthcare services for the slum-poor dwellers (78). Launching special health services such as maternal and child health (MCH), health and nutrition services, sanitation and hygiene, special eye care, and adolescent and reproductive healthcare with special health professionals among the slum dwellers can enhance access to health services. Counseling and consultation and BCC strategies and techniques can inspire and motivate slum people to use health services. Using mobile technology (e-Mobile/telehealth and telemedicine) in health services increases the popularity, usage, and access to service volume on a large scale (79). All participants unanimously believed and insisted that such initiatives and grassroots-level services should also come from the government. Big private hospitals and domestic donors align with NGOs and ensure these initiatives are implemented in all slums of Dhaka. Participants claimed and the research team also observed that only a few big NGOs such as ICDDR, B, BRAC, Radda, Shakti Foundation, and Gonoshasthaya Kendra (GSK) provided healthcare services with their static and satellite clinics to slums. Some small slums enjoy and find health facilities from big NGOs, which are relatively located in the vicinity of and at geographical disadvantage points of urban areas. FGD respondents from the Vashantek and Pallabi slums shared that they got better healthcare facilities from the NGOs than the respondents of the Rupnagar slums. A few small NGOs provided scanty health services to the slum dwellers of Rupnagar. As a result, outreached and backward slums such as Rupnagar were extremely deprived of access to these services. BRAC, Radda, GSK, and Shakti Foundation undertake initiatives and provide five days of static or satellite/outreach clinic services, affordable health packages, special health packages, referral health services for emergency or critical patients, doorstep health services, ambulance services, and 24 h CSP and CHW services through e-technology/mobile services for their slums (Vashantek and Pallabi). However, these. Still, these health services and facilities are not available in Rupnagar slums. Although some NGOs were found to be actively providing urban services to the slum communities, others were found to be preoccupied with extending their projects rather than addressing improvised people's actual needs (74–76). Most NGOs in the slums provide services on a project basis and with the support of donors as well as government registration and restrictions, so NGOs have faced challenges and drawbacks and discontinue their projects and services and tend to diminish their role due to donor's declining funds and growing government restrictions (2). NGOs emphasize counseling services in the slums rather than medication services.

### Limitations and scope of the study

The researcher and his team faced some barriers and problems while conducting the study. More or less, every slum was influenced and controlled by the local political leaders. Hence, the participants were initially disinclined and afraid of participating in interviews, but later, the researcher convinced community-supportive leaders of each slum for this study. The data collection process of this study was conducted during COVID-19, so participants did not stay in their slums, and few moved to their rural areas. As the data collection process was going on during COVID-19, most of the slum dwellers thought we visited their slum to distribute relief and donations.

### Implications

Although this study has limitations, it demonstrates an inclusive and diversified aspect of NGOs’ initiative roles and grassroots approach to healthcare services for the government, donors, and private health organizations who aspire to organize and provide health services for the urban poor slum people. Government and policymakers can undertake and launch new initiatives and grassroots approaches apart from the NGOs’ existing initiatives and grassroots level services or collaborate with existing and grassroots level services to expand and enhance healthcare services for the underserved slum dwellers.

## Conclusion

The study found that NGOs’ initiatives and grassroots approaches had a significant association and impact on using and accessing healthcare services. Initiatives including affordable or reasonable services, special health services by health professionals, BCC strategy, grassroots approaches such as doorstep services, and providing constant or 24 h services by CSPs/CHWs are mentionable for accessing health services for the slum people. Addressing these initiatives and the grassroots-level approach of NGOs is necessary to draw serious attention to other concerned policymakers for launching and triggering the same or innovative initiatives and grassroots approach for the unprivileged slum people. The study bears an inclusive scenario and insight into NGOs’ initiatives and grassroots approach for accessing health services for the slum people, and this paper paves the way for government, donors, and other concerns materializing their new projects about the disadvantaged slum people. This study also recommends and suggests that government, donors, and policymakers synchronize their initiatives and grassroots-level services with the NGOs so that such initiatives and approaches, along with their services, may easily trace, reach slum people, and help them use and access health services. Further and future studies are necessary to evaluate NGOs’ initiatives and grassroots approaches to access to health services. More novel or innovative initiatives and grassroots approaches may be explored and enhanced through this further and future works.

## Data Availability

The original contributions presented in the study are included in the article/Supplementary Material, further inquiries can be directed to the corresponding author.

## References

[1] Rahman S Tasnim F. The role of NGOs in ensuring local governance in Bangladesh: from the perception of other actors of governance. Asia Pac J Reg Sci. (2023) 7(3):1007–34. 10.1007/s41685-023-00283-w

[2] Baser S Hasnath S. The rise and fall of the NGOs in Bangladesh: what does the future hold? In: Vito B Tatjana H, editors. Global Perspectives on Non-Governmental Organizations. Ch. 4. Rijeka: IntechOpen (2022).

[3] Roy I. Contribution of NGOs for socio-economic development in Bangladesh. Sci J Bus Manag. (2017) 5:1. 10.11648/j.sjbm.20170501.11

[4] Maruf MH. GO-NGO Collaboration in Health Sector Management of Bangladesh: An Evaluation of BRAC’s Health Programme. Dhaka: BRAC (2013).

[5] HEU. Study to Define Scopes, Opportunities, Challenges, and Way Forward for Developing a Stakeholder Coordination Strategy Towards Harmonizing GO/NGO Collaboration in the Health Sector. Dhaka: Health Economic Unit (2021).

[6] Afsana K Wahid SS. Health care for poor people in the urban slums of Bangladesh. Lancet. (2013) 382(9910):2049–51. 10.1016/S0140-6736(13)62295-3 24268606

[7] Andrew C Goldsmith M. From local government to local governance—and beyond? Int Polit Sci Rev. (1998) 19(2):101–17. 10.1177/019251298019002002

[8] Bhuiyan MI Haque A. Role of NGOs in providing available and affordable health care services to the slum people in Dhaka. Clin Epidemiol Glob Health. (2024) 25:101478. 10.1016/j.cegh.2023.101478

[9] Piotrowicz M Cianciara D. The role of non-governmental organizations in the social and the health system. Przegl Epidemiol. (2013) 67(1):69–74. 151–5. 23745379

[10] Albis M Bhadra S Chin B. Impact evaluation of contracting primary health care services in urban Bangladesh. BMC Health Serv Res. (2019) 19:854. 10.1186/s12913-019-4406-5 31752843 PMC6956513

[11] MH&FW. Comprehensive Social and Behavior Change Communication Strategy. Dhaka (2016). Report No.: 984757078-7.

[12] Talukder M Yasmeen BH Nazneen R Hossain M Chowdhury I. Assessment of relevance and effectiveness of Community Health Workforce (CHW) development system in Bangladesh. North Int Med Coll J. (2015) 5(2):332–5. 10.3329/nimcj.v5i2.23129

[13] Perry H Crigler L Lewin S Glenton C LeBan K Hodgins S. A new resource for developing and strengthening large-scale community health worker programs. Hum Resour Health. (2017) 15(1):1–3. 10.1186/s12960-016-0178-8 28183351 PMC5301425

[14] Chowdhury A Perry H. NGO Contributions to Community Health and Primary Health Care: Case Studies on BRAC (Bangladesh) and the Comprehensive Rural Health Project, Jamkhed (India). Global Public Health (2020). p. 1–75.

[15] BBS. Population & Housing Census 2022. Preliminary Report. Dhaka (2022). p. 1–68.

[16] Curtis S Rahman M Barkataki S Chakraborty N. Impact of the Bangladesh Nongovernmental Organization Health Service Delivery Project. North Carolina, USA (2019). p. 1–160.

[17] NHSDP, U.-D. The NGO Health Service Delivery Project (HSDP) 2012–2018. Dhaka (2018). p. 1–94.

[18] UN. Sustainable Development Goals (SDGs): 3: Good Health and Well-Being Online: United Nations (UN) (2024). [updated 11 August 2024; cited 2024 5/4/2024]. Available from: https://bangladesh.un.org/en/sdgs/3. Online Publication

[19] Perry HB. Health for all in Bangladesh: Lessons in Primary Health Care for the Twenty-First Century. Dhaka: The University Press Limited (UPL) (2000). p. 354.

[20] Balabanova MMD Mills A. Good health at low cost’: 25 years on. What makes a successful health system? Reprod Health Matters. (2012) 20(39):212–4. 10.1016/S0968-8080(12)39614-6

[21] USAID. Bangladesh Nongovernmental Organization (NGO) Health Service Delivery Project (NHSDP). Dhaka (2016). p. 1–165.

[22] LGD. Urban Primary Health Service Delivery Project- II (UPHCSDP–II) Online: Local Government Division. Ministry of Local Government, Rural Development and Cooperatives (2024). [cited 2024 5/5/2024]. Available from: https://uphcsdp.gov.bd/background.

[23] WHO. Strategic Priorities of WHO in Bangladesh: Strategic Priority 1: Advancing Universal Health Coverage. Online: World Health Organization (WHO) (2024). [cited 2024 5/5/2024]. Available from: https://www.who.int/bangladesh/about-us/our-work.

[24] Akhter N. End Line Evaluation Of Community Health Worker Result And Implementation Approach: Good Neighbors Bangladesh. Research. Dhaka: Good Neighbours (2021). p. 1–82.

[25] Mberu BU Haregu TN Kyobutungi C Ezeh AC. Health and health-related indicators in slum, rural, and urban communities: a comparative analysis. Glob Health Action. (2016) 9(1):33163. 10.3402/gha.v9.33163 27924741 PMC5141369

[26] Mistry SK Akter F Yadav UN Hossain MB Sichel A Labrique AB Factors associated with mobile phone usage to access maternal and child healthcare among women of urban slums in Dhaka, Bangladesh: a cross-sectional study. BMJ Open. (2021) 11(4):e043933. 10.1136/bmjopen-2020-043933 33837099 PMC8043001

[27] Khan N. Maternal and child health in Bangladesh: a critical look at the policy and the sustainable development goals. Asian J Med Biol Res. (2017) 3:298. 10.3329/ajmbr.v3i3.34517

[28] DGFP, D.a. Bangladesh National Strategy for Maternal Health 2019-2030. Dhaka (2019).

[29] Nasreen H. Maternal, Neonatal, and Child Health Programmes in Bangladesh: Review of Good Practices and Lessons Learned. Dhaka (2007). p. 1–109.

[30] Feroz A Perveen S Aftab W. Role of mHealth applications for improving antenatal and postnatal care in low and middle income countries: a systematic review. BMC Health Serv Res. (2017) 17(1):704. 10.1186/s12913-017-2664-7 29115992 PMC5678803

[31] Khatun F Hanifi SMA Iqbal M Rasheed S Rahman MS Ahmed T Prospects of mHealth services in Bangladesh: recent evidence from chakaria. PLoS One. (2014) 9(11):e111413. 10.1371/journal.pone.0111413 25375255 PMC4222888

[32] Mistry SK Akter F Hossain MB Huda MN Irfan NM Yadav UN Exploring factors associated with women’s willingness to provide digital fingerprints in accessing healthcare services: a cross-sectional study in urban slums of Bangladesh. Int J Environ Res Public Health. (2021) 19(1):1–12. 10.3390/ijerph19010040 35010299 PMC8751190

[33] Rahman A Leppard M Rashid S Jahan N Nasreen HE. Community perceptions of behaviour change communication interventions of the maternal neonatal and child health programme in rural Bangladesh: an exploratory study. BMC Health Serv Res. (2016) 16(1):389. 10.1186/s12913-016-1632-y 27530405 PMC4987986

[34] Sarker B Mridha M Dasgupta S Islam N Reichenbach L. The effect of behavior change communication (BCC) interventions on maternal neonatal and child health (MNCH) knowledge in urban slums of Bangladesh No. 17 January 2012. Manoshi Working paper, icddr,b (2012).

[35] Anam ASMT Mohammad NU. Effectiveness of community clinics in primary healthcare in Bangladesh: an empirical study. Dyn Public Adm. (2021) 38(1):28–42. 10.5958/0976-0733.2021.00003.1

[36] Haque MA Choudhury N Ahmed SMT Farzana FD Ali M Naz F The large-scale community-based programme ’Suchana’ improved maternal healthcare practices in north-eastern Bangladesh: findings from a cluster randomized pre-post study. Matern Child Nutr. (2022) 18(1):e13258. 10.1111/mcn.13258 34467636 PMC8710100

[37] Sanadgol A Doshmangir L Majdzadeh R Gordeev VS. Engagement of non-governmental organisations in moving towards universal health coverage: a scoping review. Global Health. (2021) 17:129. 10.1186/s12992-021-00778-1 34784948 PMC8594189

[38] Mercer A Khan MH Daulatuzzaman M Reid J. Effectiveness of an NGO primary health care programme in rural Bangladesh: evidence from the management information system. Health Policy Plan. (2004) 19(4):187–98. 10.1093/heapol/czh024 15208275

[39] ADB. Making Health Care Affordable for the Urban Poor in Bangladesh. Dhaka: Annual Development Bank (ADB) (2017).

[40] O'Leary S. Grassroots accountability promises in rights-based approaches to development: the role of transformative monitoring and evaluation in NGOs. Account Organ Soc. (2016) 63. 10.1016/j.aos.2016.06.002

[41] Rahman R Qattan A. Health and Human Rights Linkage in Bangladesh: Stakeholders’ views from the grassroots (2020).

[42] Abed FH. Health and development: lessons from the grassroots. J Diarrhoeal Dis Res. (1996) 14(2):119–24. 8870408

[43] Tao W Chen X Gan S. How to promote grass roots medical treatment under China’s graded diagnosis and treatment policy? — from the perspective of customer value theory. Front Public Health. (2022) 10:994644. 10.3389/fpubh.2022.994644 36523586 PMC9745134

[44] Ahmed NU Alam MM Sultana F Sayeed SN Pressman AM Powers MB. Reaching the unreachable: barriers of the poorest to accessing NGO healthcare services in Bangladesh. J Health Popul Nutr. (2006) 24(4):456–66. 17591342 PMC3001149

[45] World Bank. Bangladesh Dhaka, O. Economics and Governance of Nongovernmental Organizations in Bangladesh. Dhaka: World Bank Office (2006).

[46] Mallozzi A Maxwell L Milne A Helm D Fogler J. A grassroots approach to addressing the MCH workforce crisis. J Psychosoc Rehabil Ment Health. (2022) 9(4):453–9. 10.1007/s40737-022-00278-4 35368744 PMC8960709

[47] Begum A Khan N. The role of NGOs in the development of health and population sector: a commentary. Chittagong Uni J Soc Sci. (1999) XIX(1):1–12.

[48] Patwardhan M Li S-J Miles T Dere H Khushalani D Desai S. Types of health service utilization in Mumbai slums: a community-based survey. BMC Res Notes. (2023) 16(1):289. 10.1186/s13104-023-06557-y 37875959 PMC10598993

[49] Sinha SK Shekhar R. Problems and development of slums: a study of Delhi and Mumbai. In: Sharma P Rajput S, editors. Sustainable Smart Cities in India: Challenges and Future Perspectives. Cham: Springer International Publishing (2017). p. 699–719.

[50] Chokshi M Patil B Khanna R Neogi S Sharma J Paul V Health systems in India. J Perinatol. (2016) 36:S9–S12. 10.1038/jp.2016.184 27924110 PMC5144115

[51] Tikkanen R Osborn R Mossialos E Djordjevic A Wharton G. International Profiles of Health Care Systems 2020 (2020).

[52] Ahmed K Grundy J Hashmat L Ahmed I Farrukh S Bersonda D Health, environmental and social conditions for the urban poor in the largest cities of Pakistan—policy and planning implications for urban poor health strategy. J Environ Sci Public Health. (2022) 06(0):77–95. 10.26502/jesph.96120168

[53] Ejaz I Shaikh BT Rizvi N. NGOs and government partnership for health systems strengthening: a qualitative study presenting viewpoints of government, NGOs and donors in Pakistan. BMC Health Serv Res. (2011) 11(1):122. 10.1186/1472-6963-11-122 21609480 PMC3112396

[54] Wulandari RD Hospital utilization among urban poor in Indonesia in 2018: is government-run insurance effective? BMC Public Health. (2023) 23(1):92. 10.1186/s12889-023-15017-y 36635640 PMC9835297

[55] Wulandari RD Laksono AD Mubasyiroh R Rachmalina R Ipa M Rohmah N. Hospital utilization among urban poor in Indonesia in 2018: is government-run insurance effective? BMC Public Health. (2023) 23(1):92. 36635640 10.1186/s12889-023-15017-y PMC9835297

[56] Philippines C. NGOS in the Philippines: The Role of NGOs in the Philippines in Increasing Health Access. Manila: CHILDHOPE Philippines Foundation, Inc (2022).

[57] Mary R Angela Desiree MA. Child rights for urban poor children in child-friendly Philippine cities: views from the community. Child Youth Environ. (2005) 15(2):117–37.

[58] Acosta S Goltz HH. Theory in health promotion research and practice: thinking outside the box. Patricia Goodson. Boston, MA: Jones and Bartlett. 2010. 245, pp. $78.95. Educ Stud. (2011) 47(6):583–8. 10.1080/00131946.2011.621077

[59] Andersen RM. National health surveys and the behavioral model of health services use. Med Care. (2008) 46(7):647–53. 10.1097/MLR.0b013e31817a835d 18580382

[60] Aday LA Andersen R. A framework for the study of access to medical care. Health Serv Res. (1974) 9(3):208–20. 4436074 PMC1071804

[61] Adu P. A Step-by-Step Guide to Qualitative Data Coding (2019).

[62] Braun V Clarke V. Using thematic analysis in psychology. Qual Res Psychol. (2006) 3(2):77–101. 10.1191/1478088706qp063oa

[63] Razzaque A Chowdhury MR Mustafa AHMG Mahmood SS Iqbal M Hanifi SMA Cohort profile: urban health and demographic surveillance system in slums of Dhaka (north and south) and Gazipur city corporations, Bangladesh. Int J Epidemiol. (2023) 52(5):e283–e91. 10.1093/ije/dyad080 37301741

[64] Chaudhury T Mannan I. Universal health coverage in Bangladesh: activities, challenges, and suggestions. Adv Public Health. (2019) 2019:1–12. 10.1155/2019/4954095 33281233 PMC7691757

[65] Rahman M Rana MS Rahman MM Khan MN. Healthcare service access challenges and determinants among persons with Disabilities in Bangladesh Scientific Reports. (2024) 14(1):19187. 39160270 10.1038/s41598-024-70418-2 PMC11333597

[66] Arefin K Mamun M Alam M. Health awareness and knowledge of the slum dwellers: a case study in Bangladesh. Asian Profile. (2010) 38(4):427–36.

[67] Rahaman MA Kalam A Al-Mamun M. Unplanned urbanization and health risks of Dhaka city in Bangladesh: uncovering the associations between urban environment and public health. Front Public Health. (2023) 11:1269362. 10.3389/fpubh.2023.1269362 37927876 PMC10620720

[68] Adams AM Islam R Yusuf SS Panasci A Crowell N. Healthcare seeking for chronic illness among adult slum dwellers in Bangladesh: a descriptive cross-sectional study in two urban settings. PLoS One. (2020) 15(6):e0233635. 10.1371/journal.pone.0233635 32542043 PMC7295220

[69] Khan MM Grübner O Krämer A. Frequently used healthcare services in urban slums of Dhaka and adjacent rural areas and their determinants. J Public Health (Oxf). (2012) 34(2):261–71. 10.1093/pubmed/fdr108 22241915

[70] Taufiq H. Role of NGOs in fostering equity and social inclusion in cities of Bangladesh: The Case of Dhaka (2021).

[71] Fowler P. Urban poverty in Bangladesh: slum communities, migration and social integration. Asian Aff. (2012) 43(3):504–5. 10.1080/03068374.2012.720774

[72] Rana MMP Ilina IN. Climate change and migration impacts on cities: lessons from Bangladesh. Environ Chall. (2021) 5:100242. 10.1016/j.envc.2021.100242

[73] Uddin MJ. Climate change, vulnerabilities, and migration: insights from ecological migrants in Bangladesh. J Environ Dev. (2023) 33(1):50–74. 10.1177/10704965231211589

[74] Lata LN. Neoliberal urbanity and the right to housing of the urban poor in Dhaka, Bangladesh. Environ Urban Asia. (2020) 11(2):218–30. 10.1177/0975425320938520

[75] Arefin S. Neoliberalism and the Right to the City: A Study of the Urban Poor in Dhaka City in Bangladesh (2017).

[76] Arefin S. Right to the City in the Age of Neoliberal Development: A Case Study of Two Slum Communities in Dhaka, Bangladesh. Society Register (2023) 7(2):49–70.

[77] Panday PK. Urban slum upgrading best practices in Bangladesh. In: Panday PK, editor. The Face of Urbanization and Urban Poverty in Bangladesh: Explaining the Slum Development Initiatives in the light of Global Experiences. Singapore: Springer (2020). p. 121–39.

[78] Amiresmaili M Yazdi-Feyzabadi V Heidarijamebozorgi M. Health services utilization among slum dwellers: an experience from Iran. J Educ Health Promot. (2019) 8:210. 10.4103/jehp.jehp_358_19 31807600 PMC6852376

[79] Shahen M Ahmed R Islam M. Challenges for health care services in Bangladesh: an overview. J Nurs Health Sci. (2022) 9(1):13–24. 10.9790/1959-0901011324

